# Meteorological Table

**Published:** 1812-05

**Authors:** 


### METEOROLOGICAL TABLE.

### *From March 26, to April 26, 1812.*

| | D. | Therm. | | | Barom. | | Hygrom. *Dry.——Damp.* | | | | | | Winds. | Atmos. Variation. |
|---|---|---|---|---|---|---|---|---|---|---|---|---|---|---|
| ○ | 27 | 38 | 46 | 44 | $30^{1}$ | $29^{5}$ | | | | 12 | 9 | 11 | SE.S. | C ... F ... R ... — ... in N |
| | 28 | 49 | 52 | 51 | $29^{5}$ | $^{2}$ | | | | 26 | 20 | 23 | SW...W.. | C. R .. — .. F. |
| | 29 | 52 | 53 | 49 | $29^{5}$ | — | | | | 25 | 20 | 23 | SW.. | C.. F...R..—.. .—...in N |
| | 30 | 51 | 56 | 53 | $29^{4}$ | $^{7}$ | | | | 26 | 22 | — | SW... | R. F .. R ... F. C .. |
| | 31 | 45 | 46 | 43 | $29^{8}$ | $^{5}$ | | | | 22 | 15 | 22 | E.. | C ... — ... R ... |
| | 1 | 48 | 54 | 46 | $29^{5}$ | $^{7}$ | | | | 25 | 22 | 20 | W.NW.. | C ... F ... R. C ... |
| | 2 | 49 | 52 | — | $29^{6}$ | $^{5}$ | | | | 20 | 24 | 26 | W..SW.. | F .. R ... C .... |
| | 3 | 53 | — | 49 | $29^{5}$ | $^{6}$ | | | | 27 | 21 | 22 | W..NW.. | C ... R ... F ... R ... in N |
| ◑ | 4 | 48 | 53 | 45 | $29^{6}$ | 30 | | | | 29 | 9 | 10 | NE..E. | R. F .... — .... |
| | 5 | 45 | 53 | 45 | $30^{1}$ | $^{2}$ | | | | 10 | 5 | 10 | W.N. | C .. F ... R. F ... |
| | 6 | 45 | 51 | 47 | $30^{2}$ | $^{1}$ | | | | 10 | 3 | 9 | SW.. | C .. F .. C .:. — ... |
| | 7 | 46 | 50 | 44 | $29^{9}$ | — | | | | 9 | 10 | 11 | SW..E. | C ... R .. C ... R. |
| | 8 | 42 | 45 | 40 | 30 | — | | | | 14 | — | — | S E. | R. F .... — .... |
| | 9 | 36 | 46 | 39 | $30^{1}$ | — | | | | 10 | 1 | 6 | NE. | F .. C .. F ... |
| | 10 | 39 | 44 | — | 30 | $29^{9}$ | — | 11 | 2 | 5 | — | — | NE. | F. — ... C ... |
| ● | 11 | 41 | 43 | 42 | $29^{9}$ | — | 2 | 3 | 2 | | | | SE.W. | C ... — .. — ... |
| | 12 | 42 | 50 | 43 | $29^{9}$ | — | 3 | 15 | 8 | | | | W.. | F ... — .... — .. |
| | 13 | 40 | 48 | 41 | 30 | — | 5 | 9 | 3 | | | | E.. | F .... C ... F .. |
| | 14 | 41 | 52 | 41 | 30 | $29^{9}$ | 5 | 30 | 10 | | | | E..SE.. | F .... — .... —. |
| | 15 | 41 | 53 | 43 | $29^{8}$ | $^{7}$ | 3 | 17 | 15 | | | | E.SE. | F ... — .... — .... |
| | 16 | 43 | 48 | 42 | $29^{7}$ | — | 12 | 14 | 10 | | | | NE... | C. — ... R. C .... |
| | 17 | 38 | 51 | 38 | $29^{8}$ | 30 | 10 | 35 | 19 | | | | NE... | F ... H. F .... S. in N |
| ◐ | 18 | 39 | 50 | 42 | 30 | $^{1}$ | 19 | 30 | 20 | | | | NE.. | F .... — ... — .... |
| | 19 | 40 | 50 | 43 | $30^{1}$ | — | 15 | 35 | 22 | | | | NE. | F .... C. F .... |
| | 20 | 40 | 58 | 45 | 30 | $^{1}$ | 20 | 41 | 38 | | | | NW.. | C ... F ... R. F .... |
| | 21 | 43 | 55 | 48 | $30^{2}$ | $^{1}$ | 20 | 40 | 34 | | | | N.. | F .... — .... — .... |
| | 22 | 45 | 53 | 44 | $30^{1}$ | 30 | 25 | 40 | 38 | | | | N.. | C .. F ... — .... |
| | 23 | 38 | 50 | 43 | 30 | — | 25 | 38 | 39 | | | | N.. | F .... — .... — .... |
| | 24 | 41 | 53 | 43 | 30 | — | 25 | 33 | 25 | | | | NW.. | F .... S. R ... F ... |
| | 25 | 42 | 46 | 45 | $29^{9}$ | $^{7}$ | 24 | 28 | 18 | | | | SW.. | C ... F .. R ... F. |
| ○ | 26 | 42 | 45 | 44 | $29^{6}$ | — | 13 | 18 | 16 | | | | W..S. | R ... —. F .. — .... |

The quantity of rain from the 26th of March to the 26th of April, 1 inch $\frac{56}{100}$.

In this interval the wind has blown from the East or North twenty-one days, through the greater part of which the atmosphere has been remarkably arid, and this state of siccity has been fully indicated by the hygrometer.

In the nights of the 23d and 24th, a slight congelation of water in the open air, even in London.

The diseases of this period have been chiefly rheumatic and pneumonic affections. Painful cases, under the term rheumatism, have frequently in this interval recurred about the face, and several instances of strongly marked periodical pains in that part have occurred, which have been subdued by the liquor arsenicalis. The catarrhal affection, which was remarked in the former month to have attacked horses very generally, has in this interval assumed a spasmodic character, evidenced by the sudden attack of dyspnœa, which has continued several hours in a most alarming form: we have not been informed, however, that any horse has died of this, apparently formidable, disease.

